# Wavelength Selection for Periodic Travelling Waves: An Unsolved Problem

**DOI:** 10.1007/s11538-025-01576-1

**Published:** 2026-01-14

**Authors:** Lukas Eigentler, Mattia Sensi

**Affiliations:** 1 Warwick Mathematics Institute, University of Warwick, Coventry CV4 7AL, Coventry, United Kingdom; 2 Zeeman Institute for Systems Biology & Infectious Disease Epidemiology Research, University of Warwick, Coventry CV4 7AL, Coventry, United Kingdom; 3 Department of Mathematical Sciences “G. L. Lagrange”, Politecnico di Torino, Corso Duca degli Abruzzi 24, 10129 Torino, Italy; 4 Dipartimento di Matematica, Università degli Studi di Trento, Via Sommarive 14, 38123 Povo (Trento), Italy

## Abstract

Periodic travelling waves (PTWs) are a common solution type of models describing spatio-temporal patterns in biology and ecology. Particularly in ecology, pattern formation is regarded as a resilience mechanism and an ecosystem’s ability to change its pattern wavelength is seen as a tool to adapt to environmental change. PTW solutions of corresponding mathematical models also possess this ability and typically undergo a cascade of wavelength changes in response to a gradual change in a bifurcation parameter. Extensive analysis has been conducted to develop a predictive understanding of parameter thresholds leading to wavelength changes. By contrast, theory on what determines PTW wavelength selection during a wavelength change is currently lacking and most conjectures stem from limited observations of specific simulations, or apply to special cases only. In this *unsolved problems article*, we first provide a review of how linear stability analysis and Busse balloon theory are used to predict parameter values at which PTW wavelength changes occur. On the topic of wavelength selection, we review the special case of PTWs in $\lambda$-$\omega$ systems, often used to predict wavelengths of predator-prey dynamics in the wake of an invasion front. For more general systems, we highlight that the Busse balloon theory that is so successful in determining parameter values of wavelength changes is unlikely able to provide information on PTW wavelength selection. Finally, we present new numerical trends of PTW wavelength selection during PTW-to-PTW transitions that highlight that some stable wavelengths are more frequently selected than others, and that cascades of wavelength changes can also result in extinction events despite bistability of the extinction state with PTWs. We conclude with a tentative list of potential approaches to unravel a deeper understanding of this topic. Combined, we aim to stimulate new approaches to gain more insights into the unsolved problem of PTW wavelength selection during PTW-to-PTW transitions.

## Introduction

Spatio-temporal patterns are ubiquitous in biology. They appear in spontaneous brain activity (Northoff et al. 2020a, b; Meyer-Baese et al. 2022), in gene expression, where they are fundamental to embryogenesis (Waylen et al. 2020; Arnold and Robertson 2009; Tam and Loebel 2007), and they often underpin epidemiological data (Malchow 2007; Meliker and Sloan 2011; Mason et al. 2023). Such patterns are particularly common in ecosystems (see Rietkerk and van de Koppel (2008) for an overview), ranging from coral reefs (Mistr and Bercovici 2003) to intertidal mussel beds (Gascoigne et al. 2005; van de Koppel et al. 2005) to a range of plant ecosystems (e.g., Gandhi et al. (2019); Hiemstra et al. (2002); van de Koppel and Crain (2006); Lejeune et al. (2002)). Of the latter, dryland vegetation patterns in which vegetated areas are interspersed by regions of bare soil (Gandhi et al. 2019) have received particular attention due to their widespread occurrence (Deblauwe et al. 2008; Kästner et al. 2024) and socio-economic importance (Dickovick 2014; United Nations Convention 2022). Spatio-temporal patterns in ecosystems often form due to scale-dependent feedbacks comprising long-range competition for resources and short-range facilitation (Rietkerk and van de Koppel 2008), for example competition for water and short-range facilitation due to soil modifications by dryland plants (Meron 2012; Eldridge et al. 2000; Thompson et al. 2010) and competition for algae and mutual protection due to attachment and sediment accumulation in intertidal mussel beds (Dolmer 2000; Bertness and Grosholz 1985; Côté and Jelnikar 1999).

Spatio-temporal patterns can occur in many different forms (e.g., gaps, labyrinth, spots) (Tarnita 2024). Stripe patterns are a specific type that typically occur in ecosystems with uniaxial resource flux, such as dryland plant systems on gentle slopes (Valentin et al. 1999) and intertidal mussel beds (van de Koppel et al. 2005), with stripes being perpendicular to the axis of the resource flux. This symmetry property is often exploited by mathematical models, such as the Klausmeier model for vegetation patterns (Klausmeier 1999) and the sediment-accumulation model for intertidal mussel beds (Liu et al. 2012) because it justifies the use of one-dimensional space domains oriented along the direction of resource flux. One-dimensional mathematical models, typically reaction-advection-diffusion systems or related extensions such as systems of integro-differential equations, capture spatio-temporal stripe patterns through periodic travelling wave (PTW) solutions (e.g. Sherratt (2005); Bennett and Sherratt (2018)). PTWs are solutions that are periodic in both space and time (Kopell and Howard 1973). We highlight that the focus of this paper exclusively lies on PTW solutions of reaction-advection-diffusion and related PDE models, but emphasise that PTWs also occur in integrodifferential equations (Gourley et al. 2001; Eigentler and Sherratt 2023), integrodifference equations (Kot 1992; Britton 1990), and individual based models (Sherratt 1996; Degond et al. 2022).

Pattern formation in ecological systems is regarded as an adaptation of populations to environmental stress and consequently seen as an alternative to *catastrophic tipping* (Rietkerk et al. 2021; Zelnik et al. 2015; Banerjee et al. 2025). Tipping points refer to irreversible ecosystem degradation over short timescales which can have dire consequences for humanity (Lenton et al. 2019; Armstrong McKay et al. 2022; Lenton et al. 2023). PTWs and spatial patterns more generally allow evasion of catastrophic tipping through a cascade of wavelength changes in response to increasing environmental stress (Eigentler and Sensi 2024; Zelnik et al. 2015; Bera et al. 2021; Yizhaq et al. 2005; Siteur et al. 2014; Sherratt 2016, 2013a). Mathematical models of patterned ecosystems aim to develop a comprehensive understanding of such cascades through bifurcation and stability analyses. Results are typically visualised through the *Busse balloon*, the region of stable patterns in a two-dimensional parameter space spanned by the main bifurcation parameter and one of the patterns’ emergent properties (wavelength, wavenumber, wavespeed) (Busse 1978), a concept which we review in more detail in Section 3. While the concept of the Busse balloon originates from mathematics, it has successfully been applied to empirical data as well (Bastiaansen et al. (2018)).

State-of-the-art knowledge developed through calculations of Busse balloons allows us to predict *when* (or rather *at what parameter values*) steps in the cascade of wavelength changes occur in mathematical models (Eigentler and Sensi 2024; Asch et al. 2025; Bastiaansen et al. 2020; Hamster et al. 2024; Sherratt 2015; Siero et al. 2015; Siteur et al. 2014; Bera et al. 2021; Sherratt 2016). By contrast, little is known about *how* wavelength changes occur and in particular what determines wavelength selection after an existing PTW is destabilised. While plenty of results predict wavelength selection of patterns (not exclusively PTWs) from an initially homogeneous state (see, e.g. Subramanian and Murray (2021); Zelnik and Tzuk (2017); Tarumi and Mueller (1989); Kramer et al. (1982); Weyer et al. (2023); Marquez-Lago and Padilla (2014)), there currently exists no theory on wavelength selection during PTW-to-PTW transitions. This means that mathematical models are currently unable to predict how severe ecosystem degradation processes will be when they occur.

The aim of this *unsolved problems article* is to raise awareness of the understudied and yet crucial question of wavelength selection during PTW-to-PTW transitions, and to encourage fellow mathematical biologists and analysts to tackle it. To do so, we first review modelling frameworks admitting PTWs (Section 2). We then review how Busse balloons are constructed and used to predict at what parameter values PTW wavelength changes occur (Section 3). We then provide an interlude that outlines knowledge of PTW selection for a few special cases (Section 4) before presenting new numerical data on selection principles in two models: in Section 5, we show that unlike PTW destabilisation, PTW selection likely cannot be understood by the essential spectra that underpin the Busse balloons, and in Section 6 we report trends in PTW selection data that we anticipate to act as useful inspiration for future analytical approaches to this unsolved problem.

## Modelling Frameworks

Models describing stripe patterns in ecological systems through PTWs often employ reaction-(advection)-diffusion equations. However, PTWs occur in a wide variety of model types including related PDE models (e.g. those in which diffusion operators are replaced by nonlocal terms Eigentler and Sherratt 2018; Bennett and Sherratt 2018; Eigentler and Sherratt 2023; Britton 1990). In this paper, we will illustrate the open problem of PTW wavelength during PTW-to-PTW transitions through two reaction-advection-diffusion models. Nevertheless, we stress that our discussion of previous work and open problems presented in this paper should be regarded in the wider context of PDE models admitting PTWs.

In a general form, the class of reaction-advection-diffusion models considered in this exposition can be written as

1 $$\begin{aligned} \frac{\partial {\boldsymbol{u}}}{\partial t}&= {\boldsymbol{f}}({\boldsymbol{u}}; {\boldsymbol{\alpha }}) + {\boldsymbol{N}} \frac{\partial {\boldsymbol{u}}}{\partial x} + {\boldsymbol{D}}\frac{\partial ^2 {\boldsymbol{u}}}{\partial x^2}, \quad x \in \mathbb {R}, t\ge 0. \end{aligned}$$

The vector ${\boldsymbol{u}(x,t)} = (u_1(x,t), \dots , u_n(x,t))^T \in \mathbb {R}^n$ represents model densities. The function ${\boldsymbol{f}}({\boldsymbol{u}};{\boldsymbol{\alpha }}) = (f_1({\boldsymbol{u}};{\boldsymbol{\alpha }}), \dots , f_n({\boldsymbol{u}};{\boldsymbol{\alpha }}))^T$ accounts for all non-spatial dynamics. The vector ${\boldsymbol{\alpha }} = (A, A_1, \dots , A_m) \in \mathbb {R}^{{m+1}}_+$ comprises all model parameters, and *A* denotes the main bifurcation parameter. Spatial dynamics comprise diffusion with coefficients ${\boldsymbol{D}} = \operatorname {diag}(d_1, \dots d_n)$, where $d_i >0$, and advection with speeds $\boldsymbol{N} = \operatorname {diag}(\nu _1, \dots , \nu _n)$, where $\nu _i \ge 0$.

A model of class (1) admits PTW solutions if there exist limit cycle solutions to the ODE

2 $$\begin{aligned} \boldsymbol{D}\boldsymbol{U}'' + (\boldsymbol{N}+c\boldsymbol{I})\boldsymbol{U'} + \boldsymbol{f}(\boldsymbol{U};\boldsymbol{\alpha }) = 0, \end{aligned}$$

that is obtained from (1) by introducing the travelling wave coordinate $z=x-ct$, $c \in \mathbb {R}$ and setting $\boldsymbol{U}(z) = \boldsymbol{u}(x,t)$. If $\boldsymbol{U}(z)$ is a limit cycle of (2), then $\boldsymbol{u}(x,t)$ is a PTW solution of (1).

Two example models fitting into class (1) that admit PTWs and for which cascades of wavelength changes have been reported are the *Klausmeier model* for dryland vegetation stripes (Klausmeier 1999) and the *sediment accumulation model* for stripe patterns in intertidal mussel beds (Liu et al. 2012). In the context of (1), the Klausmeier model for dryland vegetation stripes is given by

3 $$\begin{aligned} \boldsymbol{u}&= \left( \begin{array}{c} u\\ w\\ \end{array} \right) , \ {\boldsymbol{f}}(\boldsymbol{u},\boldsymbol{\alpha }) = \left( \begin{array}{c} u^2w - Bu\\ A-w-u^2w\\ \end{array} \right) , \ \nonumber \\ \boldsymbol{N}&= \operatorname {diag}(0,\nu ), \ \boldsymbol{D} = \operatorname {diag}(1,d), \ \boldsymbol{\alpha } = (A,B){,} \end{aligned}$$

after a suitable nondimensionalisation (Sherratt 2005). The model describes the dynamics of plant biomass *u* and water density *w* on a sloped terrain. The parameters represent rainfall (*A*), plant loss (*B*), speed of water flow downhill ($\nu$) and the water diffusion coefficient (*d*). Previous work has focussed on the rainfall parameter *A* as the main bifurcation parameter to address questions on how climate-change-induced changes to precipitation volumes affect striped vegetation patterns (e.g. Eigentler and Sensi (2024); Sherratt (2013a); Bastiaansen et al. (2020); Siero et al. (2015)).

A nondimensional version of the sediment accumulation model (Sherratt 2016) for intertidal mussel beds is

4 $$\begin{aligned} \boldsymbol{u}&= \left( \begin{array}{c} m\\ s\\ a\\ \end{array} \right) , \ {\boldsymbol{f}}(\boldsymbol{u},\boldsymbol{\alpha }) = \left( \begin{array}{c} \frac{\delta a m(s+\eta )}{s+1} - m \\ m-\theta s\\ 1-\varepsilon a - \frac{\beta a m(s+\eta )}{s+1}\\ \end{array} \right) , \ \nonumber \\ \boldsymbol{N}&= \operatorname {diag}(0,0,\nu ), \ \boldsymbol{D} = \operatorname {diag}(1,d,0), \ \boldsymbol{\alpha } = (\delta , \eta , \beta , \theta , \varepsilon ). \end{aligned}$$

This model describes stripe patterns of mussels (*m*) that form perpendicular to the tide axis through interactions with sediment (*s*) and their main food source, algae (*a*). The parameters are maximum mussel growth rate ($\delta$), the minimum mussel growth rate in the absence of sediment ($\eta$), sediment erosion ($\theta$), natural algae death ($\varepsilon$), algae consumption by mussels ($\beta$), the speed of tidal-driven algae flux ($\nu$), and the sediment diffusion coefficient (*d*). In model analysis, the mussel growth rate $\delta$ is typically chosen as the main bifurcation parameter (e.g. Eigentler and Sensi (2024); Sherratt (2016)). While tidal-driven algae movement would be best approximated by a near-periodic oscillation of the flux parameter about zero, the consensus view, supported by mathematical analysis (Sherratt and Mackenzie 2016), is to use models with constant advection because mussel patterns are driven by events that take place while the tide comes in; systems are largely passive while the tide flows out. 
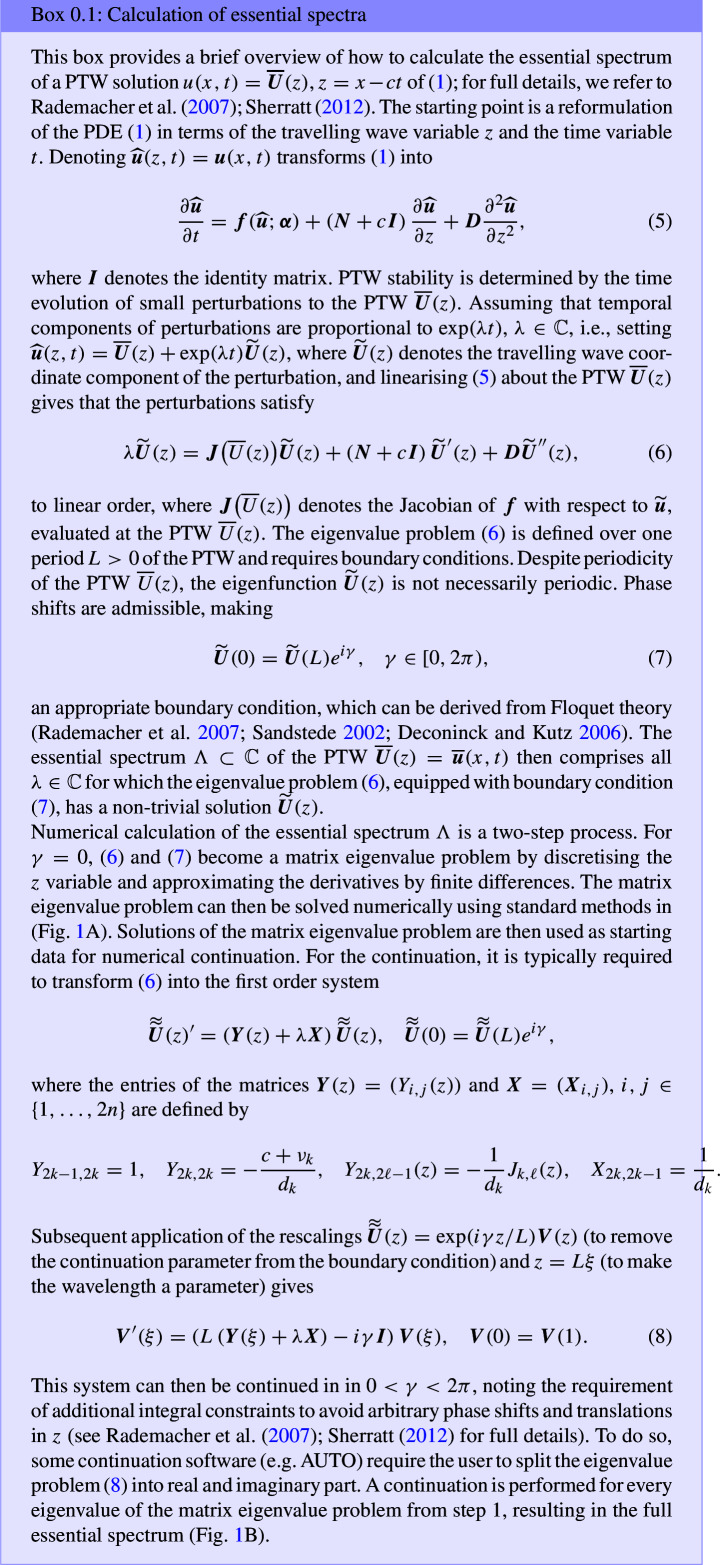


## Using Busse Balloons to Understand Destabilisation of PTWs

Solutions of models admitting PTWs and other spatial patterns typically undergo a cascade of wavelength changes as the main bifurcation parameter is varied (Bastiaansen et al. 2020; Rietkerk et al. 2021). We illustrate this through an example using a simulation of the Klausmeier model for dryland vegetation patterns (3) on a finite domain with periodic boundary conditions, visualised in Fig. 2A. Starting from a high precipitation level that supports a stable spatially uniform vegetated state, a decrease in precipitation *A* first causes the onset of PTWs via a Turing-Hopf bifurcation (Sherratt 2013c). PTW wavelength at onset can be calculated through standard linear stability analysis (Sherratt 2005). The wavelengths of solutions are robust to further decreases of the bifurcation parameter *A* for a large parameter space; modest changes in *A* affect PTW amplitude but not wavelength. Only a decrease below a threshold far from the onset point causes an abrupt transition to a solution with larger wavelength (Sherratt 2013b, 2015, 2013a) (Fig. 2A-B). These dynamics of wavelength persistence followed by an abrupt wavelength change repeat several times before the bifurcation parameter *A* decreases to small values that do not support PTW solutions. Beyond this threshold, the solution becomes a spatially uniform state representing plant extinction. Similar dynamics can be observed in the mussel model (4), albeit with fewer wavelength transitions (Fig. 3A) (Sherratt 2016; Eigentler and Sensi 2024).

**Fig. 1:**
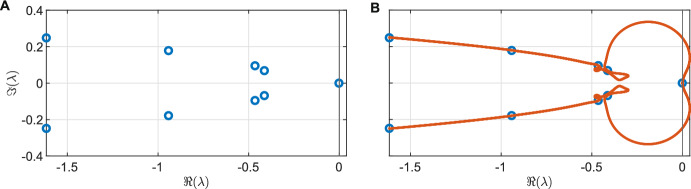
The essential spectrum for the PTW solution of the Klausmeier model (3) with $A=1.4$ and $L=20$ (green dot in Fig. 2C). **A** only shows the matrix eigenvalues calculated in the initial step of the two-step process, while **B** shows the full spectrum, still indicating the matrix eigenvalues from step 1 as blue circles

The transitions in the cascade of wavelength changes are typically irreversible due to hysteresis (Sherratt 2013a; Tarnita 2024; Rietkerk et al. 2021; Martinez-Garcia et al. 2022). Thus, the detection of parameter thresholds that lead to wavelength changes has received significant attention. Mathematically, this can be achieved by analysing pattern stability. In the context of PTWs on one-dimensional spatial domains, stability is typically determined by a solution’s *essential spectrum*, which can be calculated using numerical continuation (Rademacher et al. 2007). This suffices because the point spectra of PTWs are empty (Sandstede 2002). For a brief outline on how to calculate essential spectra, see Box 2.1. As expected from a tool determining linear stability, the essential spectrum $\Lambda \subset \mathbb {C}$ of a solution describes the behaviour of small perturbations to the solution, with the real part of the spectrum quantifying the growth rate. Thus, if $\sup _{\lambda \in \Lambda \setminus \{0\}}(\Re (\lambda )) = 0$, then the corresponding PTW is stable, and if $\max _{\lambda \in \Lambda }(\Re (\lambda )) > 0$, then the corresponding PTW is unstable. Note that the origin is excluded from the definition, because $0\in \Lambda$ always due to translation invariance of PTWs. In the special case of the origin being an eigenvalue with higher multiplicity, the corresponding PTW is unstable if $\max _{\lambda \in \Lambda }(\Re (\lambda )) > 0$, or neutrally stable otherwise. An example spectrum of an unstable PTW solution to the Klausmeier model (3) is shown in Fig. 1B, and examples of spectra of stable solutions are visualised in Fig. 4C. It is noteworthy that PTW stability can be determined analytically in special cases, for example when equations have gauge symmetry (*u* is a solution implies $e^{i\theta }u$ is a solution for all $\theta \in \mathbb {R}$) (Lloyd et al. 2025), or for $\lambda$-$\omega$ equations (see Section 4).

**Fig. 2:**
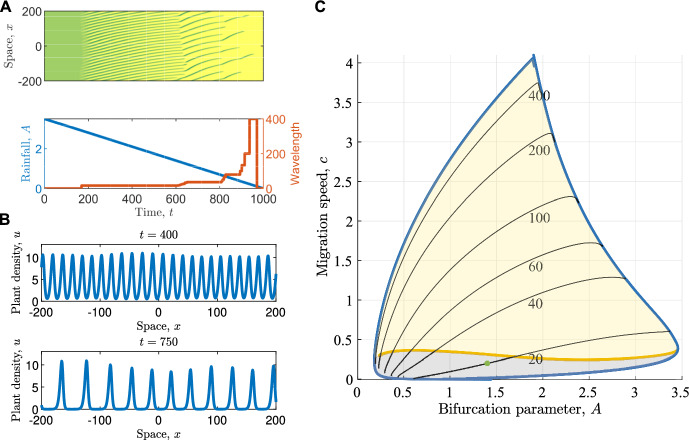
**A:** Example simulation of the Klausmeier model with varying rainfall parameter on a spatial domain $[-200,200]$ with periodic boundary conditions. The bifurcation parameter decreases from $A=3.5$ to $A=0$ at rate $3.5 \cdot 10^{-3}$. The top panel depicts the solution in time-space plane, the bottom panel shows how the bifurcation parameter (model input) and pattern wavelength (emergent property) changes over time in the simulation. Parameter values are $B=0.45$, $\nu = 182.5$, and $d=500$. **B:** Two example solutions for fixed timepoints. They correspond to $t=400$ and $t=750$ in **A**. **C:** Busse balloon for the Klausmeier model with $B=0.45$, $\nu = 182.5$, and $d=500$. Blue curves are PTW existence boundaries (homoclinic orbit and Hopf bifurcation on the left and right, respectively), the yellow curve is a stability boundary, splitting the existence region into stable (light yellow) and unstable (grey) regions, and solid annotated black curves are wavelength contours. The green dot shows the parameter values of the example spectrum shown in Fig. 1

**Fig. 3:**
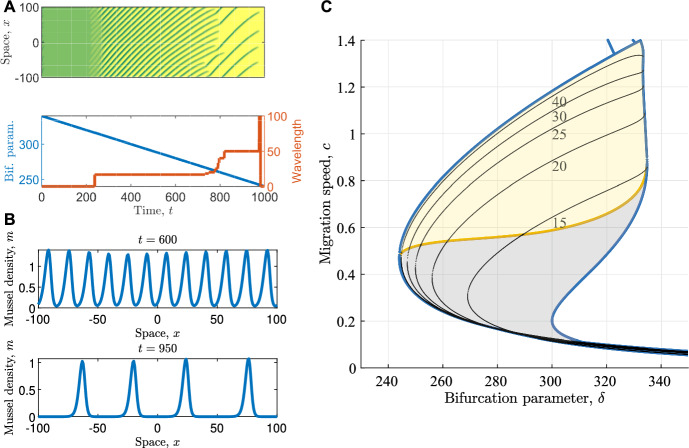
**A:** Example simulation of the mussel model with varying mussel growth rate on a spatial domain $[-100,100]$ with periodic boundary conditions. The bifurcation parameter decreases from $\delta =340$ to $\delta = 240$ at rate 0.1. The top panel depicts the solution in time-space plane, the bottom panel shows how the bifurcation parameter (model input) and pattern wavelength (emergent property) changes over time in the simulation. Parameter values are $\varepsilon = 50$, $\beta = 200$, $\eta = 0.1$, $\theta = 2.5$, $\nu = 360$, and $d=1$. **B:** Two example solutions for fixed timepoints. They correspond to $t=600$ and $t=950$ in **A**. **C:** Busse balloon for the mussel model with $\varepsilon = 50$, $\beta = 200$, $\eta = 0.1$, $\theta = 2.5$, $\nu = 360$, and $d=1$. Blue curves are PTW existence boundaries (homoclinic orbit on the left and a combination of a Hopf bifurcation and fold on the solution branches on the right, respectively), the yellow curve is a stability boundary, splitting the existence region into stable (light yellow) and unstable (grey) regions, and solid annotated black curves are wavelength contours. Note that the blue curve splitting stable and unstable solutions in the top right corner is not a stability boundary. Rather it is the location of a homoclinic solution in which stable solutions terminate. Due to the presence of folds in solution branches, unstable solutions exist above this boundary; see Sherratt (2016) for full details

Over larger parameter spaces, stability properties of PTW solutions to a model are typically summarised through stability diagrams, termed *Busse balloons* (Busse 1978). Busse balloons visualise parameter regions of stable PTWs, most commonly in a two-dimensional parameter plane spanned by the main bifurcation parameter of the model and one of the emergent PTW solution properties (wavespeed, wavelength or wavenumber). Often, as shown in the example Busse balloons depicted in Fig. 2C and Fig. 3C, these diagrams also include information on parameter regions admitting unstable PTWs, even though such regions are strictly speaking not part of the Busse balloon. In the two example models considered in this paper, the PTW existence regions are bounded above (along the axis of the bifurcation parameter) by a Hopf bifurcation of a spatially uniform steady state in the corresponding travelling wave ODE (2) and bounded below by a homoclinic orbit in the travelling wave system (essentially a PTW with period $L = \infty$). In some models, folds in solution branches, associated for example with subcritical Hopf bifurcations, mean that PTW may exist either side of Hopf and/or homoclinic loci; in these cases, the fold location forms the Busse balloon boundary (see e.g., Eigentler and Sherratt (2020)). While for some models, these boundaries can be calculated, or at least approximated analytically (e.g. Sherratt 2013c), they are most commonly determined through numerical continuation. Numerical continuation software such as AUTO-07p (Doedel et al. 2012) provide functions for two-parameter continuations of Hopf bifurcations. Homoclinic orbits are typically approximated through a two-parameter continuation of a sufficiently large wavelength contour. The PTW existence region is split into regions admitting stable and regions admitting unstable PTWs. Stability boundaries are locations in parameter space in which the essential spectrum crosses the imaginary axis. Spectra can cross the imaginary axis either through a change of curvature at the origin (*Eckhaus* or *sideband* stability boundaries), or through a pair of complex conjugate elements crossing the axis away from the origin (*Hopf* stability boundaries) (Rademacher and Scheel 2007). Both types of stability boundaries can be calculated through a numerical continuation algorithm which provides a significant computational advantage over calculating spectra on a parameter grid; for more details on these algorithms, see Sherratt (2013b). All Busse balloons and essential spectra used in this paper were calculated using AUTO-07p.

Single model realisations with a changing bifurcation parameter such as those depicted in Fig. 2A and Fig. 3A can be understood as steps through the Busse balloon. Within the Busse balloon, solutions track wavelength contours through the stability region. Once a stability boundary is crossed and the current wavelength becomes unstable, a transition to a new stable wavelength occurs. There exists anecdotal evidence of transitions to a “PTW-like” state in which amplitudes of peaks oscillate asynchronously (Bennett and Sherratt 2018; Gani and Ogawa 2016), but here we strictly focus on PTW-to-PTW transitions. It is noteworthy that these transitions do not occur instantly upon crossing a stability boundary. Instead, they are subject to a (potentially long) delay whose length is characterised by an integral of the spectrum’s maximum real part over the transient period (Eigentler and Sensi 2024).

In summary, Busse balloons are frequently used to predict under what parameter conditions a wavelength change occurs (Eigentler and Sensi 2024; Siteur et al. 2014; Rietkerk et al. 2021; Asch et al. 2025; Siero et al. 2015; Sherratt 2016). However, not much is known about the dynamics of wavelength changes themselves. In particular, we lack information on rules that determine wavelength selection. Vertical transects through a Busse balloon highlight the multistability of PTWs for any values of the bifurcation parameter within the bounds of the Busse balloon (except at fold locations of the boundaries). Thus, the Busse balloon provides a range of possible wavelengths to be selected but cannot point towards a single PTW.

## Knowledge on Special Cases

Little is known about generally applicable selection principles for PTWs. A special case for which analytical results on PTW wavelength selection exist is PTWs behind invasion fronts in $\lambda$-$\omega$ systems. These systems are regarded as a prototype system for PTWs due to the seminal work by Kopell and Howard (1973). They describe the dynamics of two real-valued densities *u*(*t*, *x*) and *v*(*t*, *x*), where $t\ge 0$ is time and $x \in D \subset \mathbb {R}$ is space. They take the general form (noting that this makes them a member of the class of models (1))

$$\begin{aligned} \frac{\partial u}{\partial t}&= \lambda (r)u - \omega (r)v + d_u\frac{\partial ^2 u}{\partial x^2}, \\ \frac{\partial v}{\partial t}&= \omega (r)u + \lambda (r)v + d_v\frac{\partial ^2 v}{\partial x^2}, \end{aligned}$$

where $r = \sqrt{u^2+v^2}$ and $\lambda (0),\omega (0)>0$. It is known that $\lambda$-$\omega$ systems admit a one-parameter family of PTW solutions given by

$$\begin{aligned} u(x,t)&= R\cos \left( \omega (R)t \pm \sqrt{\lambda (R)}x \right) , \\ v(x,t)&= R\sin \left( \omega (R)t \pm \sqrt{\lambda (R)}x \right) , \end{aligned}$$

for any amplitude $R>0$ such that $\lambda (R)>0$ (Kopell and Howard 1973). Moreover, the PTW defined by the amplitude *R* is linearly stable if (Kopell and Howard (1973))

$$\begin{aligned}4\lambda (R)\left( 1+\left( \frac{\omega '(R)}{\lambda '(R)}\right) ^2\right) + R\lambda '(R) \le 0.\end{aligned}$$

Work on which member of the one-parameter family of PTW solutions to $\lambda$-$\omega$ systems is selected has primarily considered PTWs that occur behind an invasion front, focussing on the influence of initial conditions and boundary conditions on the selection dynamics. For example, Sherratt (1994) investigated a $\lambda$-$\omega$ system with equal diffusion coefficients ($d_u=d_v$), and the specific choice of $\lambda (r) = \lambda _0 - r^p$, $\omega (r) = \omega _0-r^p$, where $\lambda _0, \omega _0, p>0$, posed on a semi-infinite spatial domain with no-flux boundary conditions at $x=0$ and $u,v \rightarrow \infty$ as $x \rightarrow \infty$, and equipped with exponentially decreasing initial data. They showed that the rate of decay of the initial condition determines which PTW is selected behind the invasion front. By contrast, switching to a homogeneous Dirichlet boundary condition $u(t,0) = v(t,0) = 0$ and restricting the analysis to $p=2$, Sherratt (2003) found through transformation into travelling wave coordinates and subsequent perturbation analysis of the resulting ODE system that the selected PTW could be determined by the PDE parameters and was independent of the initial condition. These initial results were subsequently extended to, for example, investigate systems with unequal diffusion coefficients (Merchant and Nagata 2010), nonlocal (in space) reaction terms (Merchant and Nagata 2015), obstacles in the space domain (Smith et al. 2008), and a mix of Dirichlet and Neumann boundary conditions on the respective ends of a finite domain (Smith et al. 2009).

Knowledge on PTW selection in $\lambda$-$\omega$ systems is particularly important, because more general reaction-diffusion systems can be reduced to $\lambda$-$\omega$ form (with $\lambda$ and $\omega$ as above) close to a supercritical Hopf bifurcation (Sherratt 1998) either if diffusion coefficients are equal or if the Jacobian matrix evaluated at the underlying homogeneous steady state has zero entries along the diagonal (Bennett and Sherratt 2017). Thus, $\lambda$-$\omega$ systems can be regarded as the normal form of a supercritical Hopf bifurcation in a coupled oscillatory system and provide information on PTW selection for small amplitude patterns close to such a bifurcation. There also exists a normal form reduction for subcritical Hopf bifurcations (Ermentrout et al. 1997). This results in a $\lambda$-$\omega$ system with $\lambda (r^2) = r^2(1-r^2)(r^2-a^2)$ and $\omega (r^2) = r^2(\omega _0+qr^2)$, with $a,q,\omega _0 \in \mathbb {R}$. The occurrence of different terms leads to results that are different from the supercritical case; for example, there exists a unique stable PTW in the wake of an invasion front for a system with Neumann boundary conditions (Ermentrout et al. 1997).

The motivation to study PTWs in the wake of invasion fronts is mainly motivated by predator-prey dynamics (e.g., Smith and Sherratt (2007)). The setting investigated in this paper is quite different. Rather than investigating PTW selection behind an invasion front, our interest lies in determining principles of PTW selection that hold after an existing PTW (occupying the whole domain) loses stability due to parameter changes. Moreover, models of large-scale ecosystems such as dryland vegetation patterns are typically studied on an infinite domain or a finite domain with periodic boundary conditions. We thus do not anticipate that the methods and results reviewed above are readily adaptable to this setting. Nevertheless, they, and related results, for example on PTW selection behind invasion fronts in a FitzHugh-Nagumo-type model (Carter and Scheel 2018), may act as inspiration for future analytical investigations of this important question.

## Essential Spectra are Insufficient to Determine Wavelength Selection of PTWs

In the remainder of this paper, we present some new numerical insights into PTW wavelength selection that occurs when a PTW loses stability due to a bifurcation parameter change, for example representing a climate-change-induced change in the precipitation regime underpinning dryland vegetation pattern dynamics. In this section, we highlight that despite extensive data collection and spectra calculation, we did not observe any evidence that that essential spectra (a linear tool) are capable of predicting wavelength selection. We initially hypothesised that the spectra of destabilised PTWs contain information about the newly selected wavelength; this would have created an analogue to how a dispersion relation predicts wavelength at pattern onset through determination of *the most unstable wavenumber* in a perturbation of a spatially homogeneous state. Moreover, even away from a pattern-onset bifurcation, the concept of using the most unstable wavenumber as an indicator for pattern selection has provided good approximations in the Klausmeier model, provided the system is initially close to a homogeneous state (Zelnik and Tzuk 2017). After observing no evidence to support this hypothesis (see below), we shifted our attention to the spectra of newly selected wavelengths, but again observed no evidence that they contain information about wavelength attractors (see below).

### Data Acquisition

To test whether essential spectra contain information about PTW wavelength selection, we created a dataset of wavelength changes. For both models (3) and (4), we considered three different combinations of parameter change regimes and initial conditions in numerical simulations:

**(i) Bifurcation parameter decrease at constant rate, starting from stability boundary:** In this regime, we initialised simulations with a pattern of wavelength $L>0$ on the stability boundary of the Busse balloon. We then simulated the model and let the bifurcation parameter decay at a constant rate $m>0$. For the Klausmeier model, this means $A(t) = A_{\operatorname {stab}}(L) - mt$, where $A_{\operatorname {stab}}(L)$ denotes the value of the bifurcation parameter at which the pattern of wavelength *L* loses its stability, and similar for the mussel model. In this regime, several wavelength changes may occur, but for consistency, we only recorded data of the first change. We performed these simulations for all pairs of possible starting wavelengths on a domain of length $S>0$ with periodic boundary conditions, i.e., $L \in \{S/n: n \in \mathbb {N}\}$ and logarithmically spaced $m \in [10^{-4},10^{-1}]$. This resulted in a total of 834 simulations for the Klausmeier model and 438 simulations for the mussel model. Note that the simulation regime is similar to those shown in Fig. 2 and 3, except that we do not start at a spatially uniform state. This ensures that we observe wavelength changes originating from different wavelengths; starting from a uniform solution would always lead to the same initial pattern wavelength, determined by the most unstable wavenumber of perturbations to the spatially homogeneous whose destabilisation leads to pattern onset.

**(ii) Instantaneous decrease of bifurcation parameter, starting from stability boundary:** This regime used the same initial condition as regime (i), i.e. a pattern on the stability boundary. The difference to (i) is that the bifurcation parameter was immediately set to a target value and held constant for the rest of the simulation, $A(t) \equiv A_{\operatorname {target}}<A_{\operatorname {stab}}(L)$. Simulations were performed for all pairs of possible starting wavelengths on a domain with periodic boundary conditions, i.e., $L \in \{S/n: n \in \mathbb {N}\}$ and equally spaced $A_{\operatorname {target}}\in [\overline{A_{\operatorname {min}}},A_{\operatorname {stab}}(L)]$, where $\overline{A_{\operatorname {min}}}$ denotes a lower bound of the values of the bifurcation parameter *A* featuring in the Busse balloon. The bifurcation parameter spacings were $\Delta A = 0.01$ (Klausmeier), and $\Delta \delta = 0.1$ (mussel), resulting in 2501 (Klausmeier) and 1987 (mussel) simulations.

**(iii) Constant bifurcation parameter, starting from unstable solution:** For a given wavelength $L>0$, this regime kept the bifurcation parameter constant at $A(t) \equiv A_{\operatorname {target}}$, where $A_{\operatorname {min}}(L)<A_{\operatorname {target}}<A_{\operatorname {stab}}(L)$, and $A_{\operatorname {min}}(L)$ denotes the smallest value of the bifurcation parameter for which a PTW with wavelength *L* exists. The simulation is initialised at the unstable PTW with wavelength *L* and bifurcation parameter $A_{\operatorname {target}}$. Simulations were performed for all pairs of possible starting wavelengths on a domain with periodic boundary conditions, i.e., $L \in \{S/n: n \in \mathbb {N}\}$ and equally spaced $A_{\operatorname {target}}\in [A_{\operatorname {min}}(L),A_{\operatorname {stab}}(L)]$. The bifurcation parameter spacings were $\Delta A = 0.01$ (Klausmeier), and $\Delta \delta = 0.1$ (mussel), resulting in 1846 (Klausmeier) and 696 (mussel) simulations.

For all simulations, we recorded the wavelength of the solution over time *L*(*t*) by counting the “peaks” of the solution component of interest (plant and mussel biomass, respectively). Here, a “peak” was defined as a local maximum with density at least a quarter of the global maximum (highest peak). We discounted local maxima of lower densities to ensure that peaks that disappear during a wavelength change stop contributing to our definition of wavelength once they are on a clear trajectory of disappearing. This ensures that no small amplitude oscillations artificially increase the “peak-count”.

Since pattern amplitudes depend on the bifurcation parameter and the wavelength, this relative criterion was more reliable than an absolute amplitude threshold. Moreover, simply counting peaks is a more reliable method to determine wavelengths than, for example, using the Fourier transform to determine the dominant wavenumber; see the discussion by Hamster et al. (2024) for more details.

We subsequently also recorded the initial wavelength $L_{\operatorname {start}}$, the value of the bifurcation parameter at which a wavelength change occurred $A_{\operatorname {change}}= \max \{A(t) \ \text {where} \ t: L(t) \ne L_{\operatorname {start}}\}$, and the newly selected wavelength $L_{\operatorname {new}}$. Note that wavelength changes are not instantaneous in time, but occur via a few wavelength steps during a short transient (see e.g., Fig. 2A). This is because the amplitudes of the disappearing peaks do not decrease at exactly the same rate. Thus, in parameter change regime (i), we only accepted a new wavelength $L(t) \ne L_{\operatorname {start}}$ as the newly selected wavelength $L_{\operatorname {new}}$, provided it persisted as the wavelength for at least one fifth of the time the solution spent at $L(t) = L_{\operatorname {start}}$. Visual spot checks of solutions suggested this to be a reasonable threshold. For parameter change regimes (ii) and (iii) in which the bifurcation parameter was held constant over time, we recorded $L_{\operatorname {new}}$ as the wavelength at the end of the simulation. To ensure that our simulations did not terminate during a wavelength change, we chose the simulation time sufficiently large, informed by a previous result on how long unstable wavelengths are preserved after the crossing of a stability boundary (Eigentler and Sensi 2024).

We used different domain lengths for the two models. In the Klausmeier model, we chose $S=400$, and in the mussel model $S=200$. The reduction in domain size for the mussel model was chosen because wavelength contours of large wavelengths were much closer to the edge of the Busse balloon (c.f., Fig. 2 and 3 C) and we often observed extinctions from lower wavelengths in the mussel model (see also Section 6.2. It is important to note that using a domain of length *S* with periodic boundary conditions imposes a restriction on wavelength selection; the only possible wavelengths are $\{L = S/n, n \in \mathbb {N}\}$. Thus, care needs to be taken when interpreting results from numerical simulations, for example when claiming that certain wavelengths are never selected (see Section 6): for example, while the Busse balloon shown in Fig. 2C contains a period 75 PTW for $A=2$, this solution does not exist in the context of the simulation on $x\in [-100,100]$ with periodic boundary conditions shown in Fig. 2A. Similar care is required when comparing simulation results with spectra calculations; the spectra calculations assume an infinite domain and thus lead to essential spectra consisting of (disjoint) continuous curves. On a finite domain, essential spectra comprise a discrete set of points. Given that in the case of periodic boundary conditions, this discrete set approximates well the essential spectrum of the infinite domain case (Sandstede and Scheel 2000), we use essential spectra calculated on an infinite domain throughout. For all regimes, the initial conditions were constructed using numerical continuation. There are many more options to set up numerical tests, but given the similarities between properties observed for the three chosen regimes, we refrained from further experiments.

### Spectral Properties of destabilised PTWs

We first calculated the essential spectra $\Lambda \subset \mathbb {C}$ of destabilised solutions just before the commencement of a wavelength change, i.e. the spectrum for the PTW with bifurcation parameter $A=A_{\operatorname {change}}$ and wavelength $L=L_{\operatorname {start}}$. We then compared several properties of the spectra to the newly selected wavelength $L_{\operatorname {new}}$. We considered the maximum real part of the spectrum $\max \{\Re (\lambda ): \lambda \in \Lambda \}$, the positive (recall that spectra always comprise complex conjugate elements) imaginary part of the spectrum at the maximum real part, i.e. $\Im (\lambda ^*)$ where $\lambda ^*= \arg \max \{\Re (\lambda ): \lambda \in \Lambda \}$, the positive maximum imaginary part of the part of the spectrum corresponding to growing perturbations, i.e. $\max \{\Im (\lambda ) \ \text {where} \ \lambda : \Re (\lambda )>0 \}$, and the value of the continuation parameter $\gamma$ at the location of the maximum real part of the spectrum. In both models, none of these data showed any relation to the newly selected wavelength $L_{\operatorname {new}}$ (Fig. 6).

### Surfaces Formed by Spectra of Possible Target PTWs

**Fig. 4:**
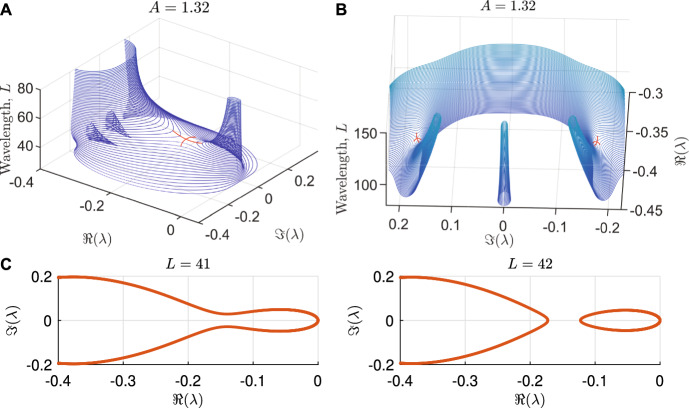
**A,B**: Saddles in the bifurcation surface (approximate stable and unstable directions are plotted as red curves). A saddle on the real axis (**A**) and a pair of complex conjugate saddles (**B**) are shown. Both visualisations are from the same spectra surface of the Klausmeier model ($A=1.32$), but note the distinct orientation and axes ranges in the two figures. The saddle on the left is on the main bifurcation surface, whereas the saddles on the right are inside the “shell” of the surface. **C**: Two spectra either side of the saddle shown in A. The other parameters are $B=0.45$, $\nu =182.5$, $d=500$

Given that no useful information could be obtained from the spectra of destabilised PTWs, we instead turned our attention to the spectra of the newly selected PTWs. For every wavelength change that occurs at $A=A_{\operatorname {change}}$, the Busse balloons for both models considered highlight that all patterns of wavelength $L>L_{\operatorname {stab}}(A_{\operatorname {change}})$, where $L_{\operatorname {stab}}(A)$ denotes the minimum wavelength giving stable PTW for a given value of the bifurcation parameter, are stable and thus possible target wavelengths (note that more generally, there could be another stability boundary restricting possible wavelengths to a finite, yet continuous interval). To investigate properties of these spectra, we created *spectra surfaces* by stacking the corresponding essential spectra for different wavelengths *L* and fixed bifurcation parameter values (Fig. 4). Our aim was to investigate the existence of an encompassing rule for wavelength selection by linking the (numerically observed) selected wavelengths to specific features of these bifurcation surfaces.

Through extensive numerical exploration, we were unable to pinpoint one specific feature of the surface as the determining factor of wavelength selection. The only “remarkable” points in these spectra surfaces are saddles, occurring both on the real axis and as pairs of complex conjugates away from the real axis. Here, a saddle corresponds to a critical wavelength value at which elements of the spectra split, creating “islands” of spectrum components disconnected from the rest of the spectrum (Fig. 4C). We remark that these saddle may appear also “inside” the surface (Fig. 4B). Moreover, couples of complex conjugate saddles are abundant *inside* the spectral surface with no apparent consequence for the dynamics. Even when restricting to saddles happening on the “outer shell” of the surface (Fig. 4A), there appears to be no regularity in how wavelength selection is associated with these saddles, i.e. connectivity properties of the spectra.

We highlight that as remarkable as these features of the spectral surface are, they are ultimately unable predict a wavelength selection. Combined, we conclude that unlike for predicting parameter values at which wavelength changes occur, the linear analysis underpinning the calculation of essential spectra is unable to shed light onto PTW wavelength selection.

## Numerical Observations of Wavelength change Dynamics After Crossing a Stability Boundary in a Busse Balloon

The lack of information on PTW wavelength selection contained in essential spectra prompted us to collect more data from our model simulations. The main goal was to detect trends in the data that could suggest further steps towards understanding wavelength selection.

**Fig. 5:**
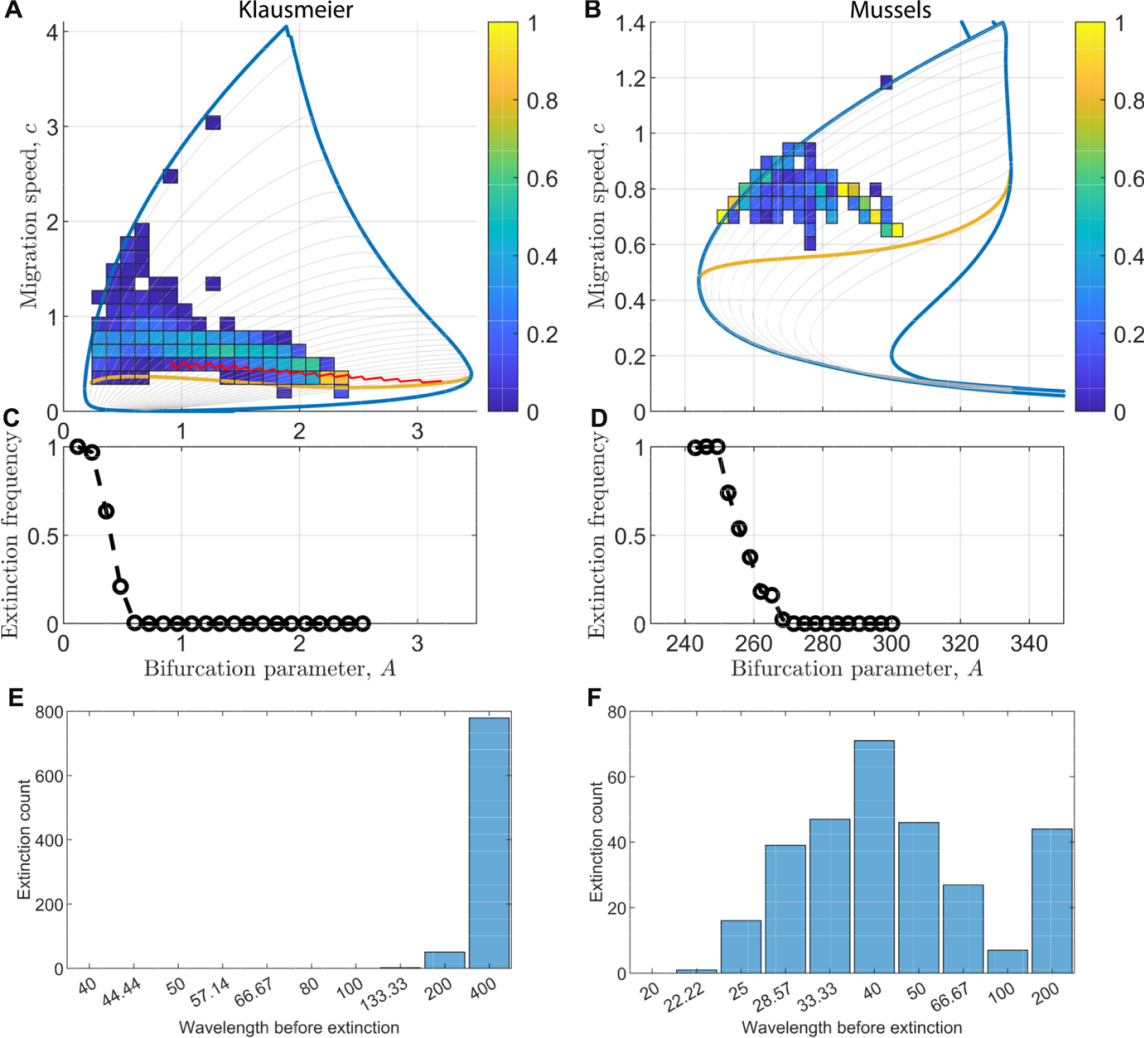
**Wavelength selection data.** **A-B:** Heatmap of wavelength selection data superimposed onto the Busse balloon for the Klausmeier model (A) and the mussel model (B). The heatmaps are compositions of 1D heatmaps for fixed bifurcation parameter, i.e. they show the relative selection frequency of *c* values for fixed bifurcation parameter *A*. Grey curves show wavelength contours of all permissible wavelengths in the simulations; wavelengths decrease from top left to right bottom of the Busse balloons. The red line in A shows the location of the wavelength corresponding to the most unstable wavenumber $L_{\operatorname {mu}}$ obtained from a linear stability analysis of the spatially uniform plant equilibrium, on a bounded domain of length 400 with periodic boundary conditions, i.e., an analysis that restricted the permissible wavelengths to $L \in \{\frac{400}{n}: n \in \mathbb {N}\}$. The equilibrium only exists for $A>2B$. For a description of the Busse balloons, see Fig. 2 and 3. **C-D:** Extinction frequency dependence on bifurcation parameter. For fixed bifurcation parameter, the proportion of simulations leading to extinction rather than a new PTWs is visualised. C: Klausmeier model; D: mussel model. **E-F:** Last wavelength before extinction. For all simulations in the parameter regime using a gradual rate of change of the bifurcation parameter, the last wavelength before extinction is recorded and their count is visualised for the Klausmeier model (E) and the mussel model (F)

### Large Regions of the Busse Balloon Remain Unselected in Numerical Simulations

For both models, we mapped wavelength selection data from all three parameter change regimes (see Section 5.1) onto the Busse balloon to visualise selection frequencies of wavelengths. This revealed that large regions of the Busse balloon remain unselected in the simulations (Fig. 5 A-B). That is, there exist many stable PTWs that are never selected in our numerical simulations. By considering vertical transects through the data mapped onto the Busse balloon, we further observed that the value of the bifurcation parameter at which a wavelength change occurred $A=A_{\operatorname {change}}$ did not uniquely determine the newly selected wavelength $L=L_{\operatorname {new}}$. This means that wavelength selection principles not only depend on the bifurcation parameter, but also on (a combination of) other system quantities, and possibly on the evolution of the PTW while losing stability (meaning, the system may retain some sort of memory).

We did not consider simulations that started from the steady state whose instability to spatially heterogeneous perturbations causes the onset of spatial patterns in the respective system. This numerical experiment was previously conducted by Zelnik and Tzuk (2017) for the Klausmeier model in the absence of advection. They found that using such initial conditions, selected wavelengths cluster around the wavelength $L_{\operatorname {mu}}$ corresponding to the wavenumber leading to the largest growth rate of small perturbations (wavenumber for which the dispersion relation derived from a linear stability analysis attains its maximum), in agreement with predictions from linear stability analysis. While this principle could not be expected to provide a universal answer (because the Busse balloon extends to regions of parameter space for which the steady state does not exist), we nevertheless compared our wavelength selection data to $L_{\operatorname {mu}}$ for the Klausmeier model with advection (3). For this model, a calculation of $L_{\operatorname {mu}}$ valid to leading order in the large parameter $\nu$ is possible (Eigentler and Sherratt 2018);

we do not anticipate the same for the mussel model due to its increased complexity and thus only focussed on the Klausmeier model. We did not observe clustering of selected wavelengths around $L_{\operatorname {mu}}$ for the Klausmeier model (Fig. 5A). We thus concluded that results from linear stability analysis of spatially homogeneous states cannot predict wavelength selection in scenarios that are initialised far from such an equilibrium state.

### Cascades of Wavelength Changes Can Stop Prematurely and Lead to Extinction

Both models considered feature a stable extinction state due to the presence of a strong Allee effect. There is thus multistability of different PTWs and an extinction state for parameter values in the range of the Busse balloon. In our dataset of wavelength changes, we indeed observed cases in which a PTW destabilised and led to extinction, even though the bifurcation parameter remained within the range of the Busse balloon. We thus investigated how the extinction frequency depended on the value of the bifurcation parameter. This revealed that no extinctions occurred provided the bifurcation parameter was sufficiently large. However, decreases of the bifurcation parameter caused an increase in the extinction frequency (Fig. 5 C-D). In both models, the Busse balloon featured values of the bifurcation parameter for which all simulations led to extinction and no new PTWs were selected, despite their stability. This re-emphasises the point made in the previous section: not all regions of the Busse balloon are selected in numerical simulations.

The simulations using the parameter change regime (i) in which the bifurcation parameter decreased at a constant rate always led to extinction eventually (possibly after a cascade of several wavelength changes) because we decreased the bifurcation parameter to low values that were outside the range of the Busse balloon. We used this data to investigate what typically is the last wavelength before extinction. The wavelength selection data (Fig. 5 A-B) showed that the largest possible wavelength (equal to domain length, thus representing a pattern with a single peak) are often selected and we thus queried whether extinction would exclusively occur via this largest wavelength or whether cascades of wavelength changes could end prematurely. This investigation revealed a stark difference between the Klausmeier model and the mussel model. In the Klausmeier model, the overwhelming majority of extinctions occurred via a cascade of wavelength changes that included the single stripe (maximum wavelength) solution (Fig. 5 E). By contrast, in the mussel model, extinctions occurred from a much larger range of wavelengths (Fig. 5 F, also c.f. Fig. 2 A and Fig. 3 A). We hypothesise that this difference can be partially explained by the spacing of wavelength contours within the Busse balloon. Given that the Busse balloons are bounded to the left (in the *A*, *c* plane) by a homoclinic orbit, the spacing between contours of wavelengths $L_1$ and $L_2$ tends to zero even as $L_1, L_2 \rightarrow \infty$, even if the difference between the wavelengths becomes arbitrarily large, i.e., $|L_1 - L_2| \rightarrow \infty$ as $L_1, L_2 \rightarrow \infty$. For fixed wavelength, the proximity of the contour to the homoclinic orbit is model (and parameter) dependent: in the Klausmeier model, contours are further from the homoclinic orbit than in the mussel model (c.f. Fig. 2 C and Fig. 3 C). If a PTWs whose wavelength contour is close to the homoclinic orbit destabilises, we hypothesise it to be more at risk of a transition to extinction because of a delayed loss of stability phenomenon. Unstable PTWs can persist as transients for a time period that is characterised by the integral of the maximum real part of the PTW essential spectrum over time (Eigentler and Sensi 2024). If the bifurcation parameter decreases at a constant rate, this can lead to situations in which the destabilised PTW persists until the bifurcation parameter leaves the range of the Busse balloon. This is more likely to occur if the wavelength contour is close to the homoclinic orbit, since the homoclinic solution provides a lower bound for the Busse balloon with respect to the bifurcation parameter. In these cases, extinction is the only possible long-term outcome. However, given that we reported above that extinction can occur even if the bifurcation parameter remains within the range of the Busse balloon, we highlight that other currently unknown processes are likely to affect extinction dynamics also.

## Outlook

As evidenced by the vast body of literature on the topic, PTWs are a common solution type of PDEs and other mathematical models describing spatio-temporal patterns in ecology and biology. Thus, understanding wavelength changes of PTWs, that is the transition from one PTW to another after a destabilisation event, is of utmost importance in many contexts. The most common examples stem from patterns in ecological systems, such as dryland vegetation patterns and intertidal mussel beds. Due to anthropogenic climate change, ecological systems experience environmental change at an unprecedented rate, typically towards harsher conditions.

Recent modelling has shown that adaptive wavelength changes prevent catastrophic tipping, i.e. full ecosystem degradation, after crossing a threshold and systems’ ability to do so is thus regarded as a resilience mechanism (Rietkerk et al. 2021). As a consequence, a lot of past research has focussed on predicting *when*, or rather *at what parameter values* PTW wavelength changes occur, for example by calculating the Busse balloon of the system of interest (see Section 3). However, questions of *how* PTW adaptively change their wavelength, and in particular what determines wavelength during PTW-to-PTW transitions remains understudied. Plenty of numerical observations of wavelength changes are available in the literature, and analytical results exist for special cases (see Section 4). However, no unifying theory and analysis results of PTW wavelength selection are currently available. In this work, we provided an overview of this challenging mathematical problem, an abundance of numerical observations and considerations, and we shall conclude it with a list of speculative suggestions for future research.

In previous work (Eigentler and Sensi 2024), we showed that the history of a solution, while it moves in the stable region of the Busse balloon, has no impact on its loss of stability that occurs after crossing a stability boundary. Hence, this part of the dynamics is unlikely to be important in the selection of a new wavelength either. On the contrary, we discovered that the dynamics occurring between PTW destabilisation and the onset of wavelength changes determine when a wavelength change occurs. We thus anticipate that this transient phase is also key in determining PTW wavelength selection. However, while linear analysis (i.e. information contained in essential spectra) was able to determine when PTW wavelength changes occur, the evidence presented in this paper highlights that essential spectra are unlikely able to provide insights into PTW selection (Section 5). Indeed, we observed that PTWs which undergo a wavelength change at (roughly) the same value of the bifurcation parameter do not necessarily select the same new wavelength upon re-acquiring stability (Fig. 5). This fact implies that a simple interpretation of “basins of attraction” of equilibria (in this case, of PTW solutions with specific wavelengths) does not apply here, and strengthens our previous consideration that instead the history of the destabilisation process influences PTW wavelength selection. In a way, these solutions seem to retain a “memory” of the unstable part of the bifurcation process. What still remains to be determined is which quantity, or quantities, one needs to keep track of in order to solve this problem (if any such quantity even exists).

The work of Asch et al. (2025), which resolves the wavelength selection problem for small wavenumber changes in the complex Ginzburg-Landau equation, a prototype system for PTW, by calculating spatio-temporal resonances, is an important step in this direction. This ought to be further explored for more general models and more general wavelength transitions. In particular, attention needs to be given to an extension of these results to systems defined on infinite domains, as the current analysis relies on the formulation of the problem on a finite domain. Moreover, Asch et al. (2025)’s analysis employs a change in the bifurcation parameter at a constant rate only, which is a restriction that is suggested to be removed in future work. Similarly, tools from other analyses of special cases (Section 4) or related topics, such as “stripe splitting” in growing domains (Ueda and Nishiura 2012; Krause et al. 2023) could be utilised in future work.

Both models studied in this work contain parameters that differ by orders of magnitude, which make them both perfect cases for the use of tools from Geometric Singular Perturbation Theory (GSPT). Promising results have been obtained by the application of these techniques in the past, see e.g. Kramer et al. (1982); Doelman et al. (2012, 2018); Weyer et al. (2023); however, to the best of our knowledge, the selection criterion of wavelength after loss of stability in PTWs is not amongst them. Many of these results concern systems of ODEs derived from the corresponding systems of PDEs by considering travelling waves coordinates. In this setting, PTWs naturally arise as families of limit cycles in phase space foliating the region contained within a homoclinic orbit; see e.g. Doelman et al. (2018), Fig. 6. Indeed, the assumptions on the parameters which are needed to derive these results are, in a general setting, somewhat restrictive; however, these can often be *sufficient* rather than *necessary*, and the predicted dynamics may be observed even when the required separation is relaxed. We believe that GSPT might still provide meaningful insight into this open problem of wavelength selection, and should be explored further.

**Fig. 7:**
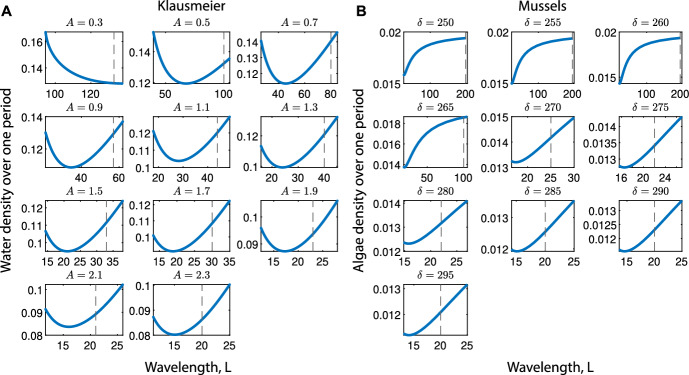
Comparison between the density of the fast diffuser (water in the Klausmeier model (**A**) and algae in the mussel model (**B**)) over one PTW period (normalised by period) and the most frequently selected wavelength. Each panel shows how the density of the fast diffuser varies with PTW wavelength (blue curve) for different values of the main bifurcation parameter. The black dashed line indicates the most frequently selected wavelength for this value of the bifurcation parameter. Note that the most frequently selected wavelength does not coincide with the minimum in the fast diffuser density. Also note the changes in the horizontal and vertical axes across both parts)

While energy minimisation principles are only applicable to closed systems (mass conservation), it may be possible that PTW wavelength selection is driven by a different minimisation law. In Turing-type systems, such as the Brusselator or the Schnakenberg model, the selected wavelength (starting from a uniform state with small perturbation added) typically minimises the mass of the fast density (Subramanian and Murray 2021). We have tested this hypothesis for the Klausmeier model and the sediment accumulation model for intertidal mussel beds. For this, we calculated the (normalised by wavelength) total mass of the water and algae densities respectively, over one wavelength across the Busse Balloon and compared it with our numerical data on wavelength selection. We did not find agreement between PTW wavelength selection and mass minimisation (Fig. 7). We nevertheless hypothesise that some other, yet to be determined, quantity is minimised at the selected wavelength and suggest further investigation. Given our results that PDE parameter values cannot uniquely determine the selected wavelength (Section 6.1), this quantity likely contains information about the original wavelength or the history of the solution.

In this *unsolved problem article*, we collated the plethora of information available on PTW selection and presented new comprehensive numerical data on the phenomenon. We argue that both the important biological questions and consequences associated with PTW wavelength selection and its fascinating underlying mathematical structure means that the mathematical biology and theoretical ecology community would benefit from a deeper and more detailed exploration. We thus encourage all researchers with expertise in the analysis of partial differential equation models to embark on providing more insights to this challenging open problem.

## Figures and Tables

**Fig. 6:**
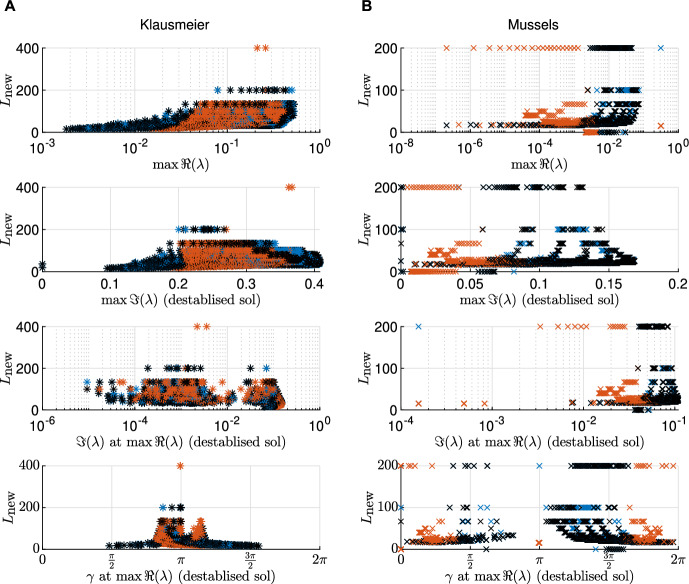
Spectra properties of destabilised PTW before a wavelength change plotted against the newly selected wavelength $L_{\operatorname {new}}$ in the Klausmeier model (**A**) and the mussel model (**B**). In both **A** and **B**, the top row shows the maximum real part of the spectrum before the wavelength change, the second row shows the maximum imaginary part in the region where the real part is positive, the third row shows the (positive) imaginary part of the spectrum at the location of the maximum in the real part, and the bottom row shows the value of the continuation parameter $\gamma$ at the location of the maximum in the real part. Red, blue and black markers correspond to parameter change regimes (i), (ii) and (iii), respectively. Parameters for the Klausmeier model are $B=0.45$, $\nu =182.5$, $d=500$, and parameters for the mussel model are $\varepsilon = 50$, $\beta = 200$, $\eta = 0.1$, $\theta = 2.5$, $\nu = 360$, $d=1$

## Data Availability

Solution data and spectra data has been made available in a BioStudies repository (Eigentler and Sensi 2025).
